# The Composite Immune Risk Score predicts overall survival after allogeneic hematopoietic stem cell transplantation: A retrospective analysis of 1838 cases

**DOI:** 10.1002/ajh.26792

**Published:** 2023-01-02

**Authors:** Yigeng Cao, Xiaowen Gong, Yahui Feng, Mingyang Wang, Yu Hu, Huilan Liu, Xueou Liu, Saibing Qi, Yanping Ji, Fang Liu, Huaiping Zhu, Wenwen Guo, Qiujin Shen, Rongli Zhang, Ningning Zhao, Weihua Zhai, Xiaoqiang Song, Xin Chen, Liangquan Geng, Xia Chen, Xuetong Zheng, Qiaoling Ma, Baolin Tang, Jialin Wei, Yong Huang, Yuanyuan Ren, Kaidi Song, Donglin Yang, Aiming Pang, Wen Yao, Yi He, Yue Shang, Xiang Wan, Wei Zhang, Song Zhang, Guangyu Sun, Sizhou Feng, Xiaofan Zhu, Mingzhe Han, Zhen Song, Ye Guo, Zimin Sun, Erlie Jiang, Junren Chen

**Affiliations:** ^1^ State Key Laboratory of Experimental Hematology, National Clinical Research Center for Blood Diseases, Haihe Laboratory of Cell Ecosystem, Institute of Hematology & Blood Diseases Hospital Chinese Academy of Medical Sciences & Peking Union Medical College Tianjin China; ^2^ Tianjin Institutes of Health Science Tianjin China; ^3^ Department of Hematology The First Affiliated Hospital of University of Science and Technology of China Hefei China; ^4^ Blood and Cell Therapy Institute, Division of Life Sciences and Medicine, Anhui Provincial Key Laboratory of Blood Research and Applications University of Science and Technology of China Hefei China; ^5^ Anhui Medical University Hefei China; ^6^ Department of Hematology Affiliated Hospital of Jiangsu University Zhenjiang China

## Abstract

There has been little consensus on how to quantitatively assess immune reconstitution after hematopoietic stem cell transplantation (HSCT) as part of the standard of care. We retrospectively analyzed 11 150 post‐transplant immune profiles of 1945 patients who underwent HSCT between 2012 and 2020. 1838 (94.5%) of the cases were allogeneic HSCT. Using the training set of patients (*n* = 729), we identified a composite immune signature (integrating neutrophil, total lymphocyte, natural killer, total T, CD4^+^ T, and B cell counts in the peripheral blood) during days 91–180 after allogeneic HSCT that was predictive of early mortality and moreover simplified it into a formula for a Composite Immune Risk Score. When we verified the Composite Immune Risk Score in the validation (*n* = 284) and test (*n* = 391) sets of patients, a high score value was found to be associated with hazard ratios (HR) of 3.64 (95% C.I. 1.55–8.51; *p* = .0014) and 2.44 (95% C.I., 1.22–4.87; *p* = .0087), respectively, for early mortality. In multivariate analysis, a high Composite Immune Risk Score during days 91–180 remained an independent risk factor for early mortality after allogeneic HSCT (HR, 1.80; 95% C.I., 1.28–2.55; *p* = .00085). In conclusion, the Composite Immune Risk Score is easy to compute and could identify the high‐risk patients of allogeneic HSCT who require targeted effort for prevention and control of infection.

## 1 INTRODUCTION

Hematopoietic stem cell transplantation (HSCT) presents a unique opportunity to study how the immune system restarts from a “blank slate.” Immune reconstitution after HSCT is highly variable among patients, and this variability influences prognosis. CD4^+^ T cell count has been proposed as a clinical predictor for prognosis after HSCT.^1^, ^2^, ^3^, ^4^, ^5^ Other researchers have reported that earlier reconstitution of natural killer (NK) cells^6^, ^7^, ^8^ and hematogones^9^ or earlier attainment of a “healthy” multivariate immune status^10^ could predict better survival or lower rate of complications after transplant. We also know that both the stem cell source^11^ and the conditioning regimen^4^ could influence the speed of immune reconstitution.

Due to the high heterogeneity of patients and overall small sample sizes, however, it is difficult to summarize all the published literature regarding post‐transplant immune reconstitution. Previous studies usually did not conduct an independent validation of their major results and in addition were often limited by small population size (less than 200 patients)^1^, ^3^, ^6^, ^8^, ^9^, ^10^, ^12^, ^13^, ^14^, ^15^, ^16^, ^17^, ^18^, ^19^, ^20^, ^21^, ^22^, ^23^, ^24^ or short duration of immune profiling (1 year or less).^3^, ^4^, ^6^, ^7^, ^8^, ^9^, ^11^, ^12^, ^14^, ^15^, ^17^, ^19^, ^21^, ^22^, ^23^, ^25^ Moreover, aside from some exceptions,^10^, ^26^, ^27^ researchers often did not evaluate multiple immune variables holistically when assessing a patient's immune reconstitution; in other words, there has been no consensus on how to assign a score to a patient's multi‐time‐point and multivariate immune data.^5^

In this study, we focused on the “cell count recovery” aspect of immune reconstitution and retrospectively analyzed 1945 patients who received HSCT between 2012 and 2020 at two medical centers in China. 1838 (94.5%) of the cases were allogeneic HSCT (allo‐HSCT). We utilized unsupervised machine learning to visualize the time course of immune reconstitution. Furthermore, we identified and validated a composite immune signature during days 91–180 post‐transplant for predicting early mortality in allo‐HSCT.

## 2 METHODS

### 2.1 The Systems Kinetics of Immune Reconstitution Tianjin patient cohort

We have retrospectively compiled post‐transplant multidimensional time‐series immune data of 1245 adult patients of HSCT at the Institute of Hematology, Chinese Academy of Medical Sciences (IHCAMS) between 2012 and 2020, 76 pediatric patients at the IHCAMS between 2017 and 2020, and 624 patients at the First Affiliated Hospital of University of Science and Technology of China (FAHUSTC) between 2015 and 2019—hereafter referred to as the “SKIRT” (Systems Kinetics of Immune Reconstitution Tianjin) dataset. The cases that experienced transplant failures, received HSCT treatment more than once, or did not have immune profiling data after day 60 post‐transplant were excluded from the analysis. The patient selection flowchart is available in Appendix p. 3.

### 2.2 Ethics approval statement

The “NICHE‐SKIRT” project (that is, the retrospective analysis of the IHCAMS patients in SKIRT) was approved by the IHCAMS Clinical Research Academic Committee on January 11, 2021 (IIT2021007) and by the IHCAMS Ethics Committee on February 7, 2021 (IIT2021007‐EC‐1). The data curation effort for the FAHUSTC patients started in May 2021. The FAHUSTC Ethics Committee approved the inclusion of the FAHUSTC patients into the SKIRT dataset (2022‐RE‐070) on May 5, 2022.

### 2.3 Clinical definitions

Acute graft‐versus‐host disease (aGVHD) was defined as a manifestation of aGVHD symptoms and signs within 100 days of transplantation. To unify the definition and grading of aGVHD, all the aGVHD cases were re‐graded according to the MAGIC criteria.^28^ “Severe aGVHD” was defined as grade III–IV aGVHD.

“Infection” was defined in two ways (adapted from the definitions described in Storek et al. (2001)^12^): “definite infection” and “clinical infection.” “Definite infection” was defined as meeting at least one of the following criteria: the culture‐confirmed presence of bacteria or fungi in a sample collected from a sterile site (for example, blood withdrawal and peritoneal fluid aspiration); polymerase chain reaction‐confirmed viremia at ≥5000 copies/ml for the cytomegalovirus or ≥10 000 copies/ml for the Epstein–Barr virus; or body temperature ≥38°C with culture‐confirmed presence of pathogens from a non‐sterile site. If the pathogen was isolated from the skin, mouth, or perianal region, manifestation of relevant signs of infection was also required for the patient to be classified as having “definite infection.” If a patient did not meet the criteria for “definite infection” and yet the body temperature was ≥38°C with documentation of infection diagnosis entered by the attending physician, we defined the patient to have had “clinical infection.” For pneumonia, a diagnosis of “clinical infection” required evidence from chest computed tomography. A total of 1718 infectious episodes post‐transplant were identified in the SKIRT cohort of patients, with 51.7% (889 of 1718) of the episodes being “definite infections.”

“Relapse” was defined as blasts ≥5% in bone marrow aspirations, appearance of pathological blasts in peripheral blood, or extramedullary recurrence in the neoplasm cases. “Relapse‐related mortality” was defined as unremitted relapse at the time of death or death caused by complications of treatment for relapse. All the other deaths were classified as “non‐relapse mortality” (NRM). Among the cases of NRM, presence of uncured infection at the time of death was classified as “infection‐related mortality.” The last day of clinical outcome follow‐up was May 16, 2022 for the IHCAMS patients and April 25, 2022 for the FAHUSTC patients.

### 2.4 The post‐transplant immune profiles

An “immune profile” in this study comprised 20 clinical features of the peripheral blood that included six features (white blood cell, neutrophil, eosinophil, basophil, monocyte, and lymphocyte counts) from complete blood counts, eight features (monocyte and lymphocyte percentages in nucleated cells; NK, total T, CD4^+^ T, CD8^+^ T, regulatory T (Treg), and B cell percentages in lymphocytes) from multiparameter flow cytometry (using monoclonal antibody combinations CD3/CD4/CD8/CD16/CD19/CD25/CD56/CD127), and six features (NK, total T, CD4^+^ T, CD8^+^ T, Treg, and B cell counts) that were derived from the other features in the “immune profile.” (For instance, “NK cell count” was calculated as the product of “lymphocyte count” and “NK cell percentage in lymphocytes.”)

For each of the 20 clinical features included in the immune profiles, we linearly rescaled the raw values across the 11 150 immune profiles into numbers between 0 (the lowest value) and 1 (the highest value). The t‐distributed stochastic neighbor embedding algorithm was then used to project all the immune profiles onto a two‐dimensional “phase space.”

To calculate the average trajectory location of a set of patients at time t, the average location of all the immune profiles collected during $\left(t-\frac{\mathrm{\delta t}}{2},t+\frac{\mathrm{\delta t}}{2}\right)$ from these patients was used as the estimate. For 0<t≤90 days, we set δt=30 days; for 90<t≤360 days, we set δt=90 days; and for t>360 days, we set δt=180 days. The “average trajectory” was calculated as the mean of means across 100 bootstrap samples.

To quantify the phenotypic gradients along the horizontal and vertical axes, we modeled a clinical feature φ by the least‐squares linear regression $\varphi \left(x,y\right)=c+\alpha \left(x\right)+\beta \left(y\right)+\varepsilon$, where $\varphi \left(x,y\right)$ was the observed value of φ at the location $\left(x,y\right)$ in the phase space, $\alpha \left(x\right)$ was the contribution of the horizontal axis to φ, $\beta \left(y\right)$ was the contribution of the vertical axis to φ, c was a constant term, and ε was residual.

### 2.5 Discovering a “high‐risk” composite immune signature for early mortality in allo‐HSCT

To guard against overfitting, the allo‐HSCT recipients in SKIRT were divided into three distinct, non‐overlapping subsets: the 729 patients before (excluding) January 1, 2019, at the IHCAMS constituted the training set, the 485 patients after January 1, 2019, at the IHCAMS constituted the validation set, and all the 624 patients at the FAHUSTC constituted the test set.

We first overlaid a 121‐by‐121 grid across the two‐dimensional phase space of immune status. At any given time t after transplant, each grid point induced a binary classification of the training‐set patients who had available immune profiling data around t: those who were near the grid point, and those who were not. We then performed Cox regression on this binary classification against all‐cause death. A grid point whose *p* value was <.01 was marked as an “anchor point.” We repeated this procedure for all the grid points until we identified all the anchor points. A patient that was near an anchor point at time t was deemed to be carrying a “‘high‐risk’ composite immune signature.” To further simplify the “high‐risk” composite immune signature into a formula that was easy to compute at the clinic, we fit a logistic regression model using forward‐and‐backward stepwise variable selection; this resultant regression model was called the “Composite Immune Risk Score.”

To avoid over‐fitting, the set of “anchor points” and the logistical model for the Composite Immune Risk Score were both calculated solely based on the training set, that is, the 729 allo‐HSCT cases before January 1, 2019 at the IHCAMS. To better estimate the prognostic value of the Composite Immune Risk Score and also to assess how sensitive the model was to changes in the patient cohort, we conducted additional verification of the Composite Immune Risk Score in the IHCAMS validation set and the FAHUSTC test set. To correct for potential confounding factors caused by biased data or unbalanced data, we used multivariate Cox regression to assess if the Composite Immune Risk Score was an independent predictor for early mortality when other factors such as patent age, primary disease, transplant type, etc. were included as the covariates. Moreover, subgroup analysis was conducted for the multivariate Cox regression to examine if the Composite Immune Risk Score remained an independent predictor for early mortality when the patient population was limited to adult or pediatric cases.

## 3 RESULTS

The basic information about the 1945 patients is summarized in Table 1. SKIRT contained 11 150 post‐transplant immune profiles from the 1945 individuals, each of whom had ≥1 immune profiles after 60 days post‐transplant (median, 4 immune profiles after 60 days; range, 1–18; 25th percentile (Q1), 2; 75th percentile (Q3), 6). 585 (30.1%) of the 1945 patients had immune profiling data after 2 years post‐transplant (Appendix, p. 4). The median duration of post‐transplant immune profiling was 1.3 years (range, 0.2–7.7; Q1, 0.7; Q3, 2.1), and the median duration of clinical outcome follow‐up was 2.7 years (range, 0.2–8.9; Q1, 1.4; Q3, 4.3).

**TABLE 1:** Basic characteristics of the 1945 patients in Systems Kinetics of Immune Reconstitution Tianjin (SKIRT)

	IHCAMS (*n* = 1321)			FAHUSTC (*n* = 624)		*p* value
	Auto‐HSCT (*n* = 107)	UCB allo‐HSCT (*n* = 35)	Non‐UCB allo‐HSCT (*n* = 1179)	UCB allo‐HSCT (*n* = 526)	Non‐UCB allo‐HSCT (*n* = 98)	
Patient age in years, median (range)	33 (12–63)	8 (2–15)	35 (2–67)	13 (1–66)	30 (4–61)	<.0001^a^
Patient sex, *n* (%)						.213 ^b^
Male	69 (64.5)	22 (62.9)	669 (56.7)	292 (55.5)	48 (49.0)	
Female	38 (35.5)	13 (37.1)	510 (43.3)	234 (44.5)	50 (51.0)	
Patient race, *n* (%)						<.0001^b^
Han	100 (93.5)	31 (88.6)	1108 (94.0)	521 (99.0)	98 (100.0)	
Manchu	3 (2.8)	0 (0.0)	27 (2.3)	0 (0.0)	0 (0.0)	
Mongol	2 (1.9)	1 (2.9)	19 (1.6)	0 (0.0)	0 (0.0)	
Hui	0 (0.0)	1 (2.9)	13 (1.1)	2 (0.4)	0 (0.0)	
Others	2 (1.9)	2 (5.7)	12 (1.0)	3 (0.6)	0 (0.0)	
Primary disease, *n* (%)						<.0001^b^
ALL/AML	103 (96.3)	24 (68.6)	741 (62.8)	396 (75.3)	47 (48.0)	
MDS	0 (0.0)	3 (8.6)	211 (17.9)	31 (5.9)	17 (17.3)	
Other neoplasms	3 (2.8)	0 (0.0)	68 (5.8)	33 (6.3)	7 (7.1)	
BMF	1 (0.9)	8 (22.9)	158 (13.4)	58 (11.0)	26 (26.5)	
Other non‐neoplasms	0 (0.0)	0 (0.0)	1 (0.1)	8 (1.5)	1 (1.0)	
Transplantation type, *n* (%)						<.0001^b^
Auto‐HSCT	107 (100.0)	0 (0.0)	0 (0.0)	0 (0.0)	0 (0.0)	
MSD non‐UCB allo‐HSCT	0 (0.0)	0 (0.0)	638 (54.1)	0 (0.0)	91 (92.9)	
Haplo non‐UCB allo‐HSCT	0 (0.0)	0 (0.0)	436 (37.0)	0 (0.0)	7 (7.1)	
MUD non‐UCB allo‐HSCT	0 (0.0)	0 (0.0)	89 (7.5)	0 (0.0)	0 (0.0)	
MMUD non‐UCB allo‐HSCT	0 (0.0)	0 (0.0)	16 (1.4)	0 (0.0)	0 (0.0)	
UCB allo‐HSCT	0 (0.0)	35 (100.0)	0 (0.0)	526 (100.0)	0 (0.0)	
Stem cell source, n (%)						<.0001^b^
PB	104 (97.2)	0 (0.0)	1104 (93.6)	0 (0.0)	68 (69.4)	
BM	1 (0.9)	0 (0.0)	3 (0.3)	0 (0.0)	0 (0.0)	
PB + BM	1 (0.9)	0 (0.0)	72 (6.1)	0 (0.0)	30 (30.6)	
UCB	1 (0.9)	35 (100.0)	0 (0.0)	526 (100.0)	0 (0.0)	
Donor‐patient relationship, n (%)						<.0001 ^b^
Self	107 (100.0)	0 (0.0)	0 (0.0)	0 (0.0)	0 (0.0)	
Parents/children	0 (0.0)	0 (0.0)	304 (25.8)	0 (0.0)	1 (1.0)	
Siblings	0 (0.0)	1 (2.9)	757 (64.2)	0 (0.0)	97 (99.0)	
Other relatives	0 (0.0)	0 (0.0)	13 (1.1)	0 (0.0)	0 (0.0)	
Unrelated	0 (0.0)	34 (97.1)	105 (8.9)	526 (100.0)	0 (0.0)	
HLA mismatch, *n* (%)						<.0001^b^
None	107 (100.0)	2 (5.7)	739 (62.7)	11 (2.1)	91 (92.9)	
1 locus	0 (0.0)	9 (25.7)	36 (3.1)	22 (4.2)	1 (1.0)	
2 loci	0 (0.0)	9 (25.7)	20 (1.7)	63 (12.0)	3 (3.1)	
3 loci	0 (0.0)	4 (11.4)	55 (4.7)	122 (23.2)	0 (0.0)	
4 loci	0 (0.0)	6 (17.1)	89 (7.5)	151 (28.7)	1 (1.0)	
5 loci	0 (0.0)	4 (11.4)	226 (19.2)	95 (18.1)	2 (2.0)	
6 loci	0 (0.0)	1 (2.9)	14 (1.2)	54 (10.3)	0 (0.0)	
7 loci	0 (0.0)	0 (0.0)	0 (0.0)	8 (1.5)	0 (0.0)	
Transplantation year, *n* (%)						<.0001^b^
2012	10 (9.3)	0 (0.0)	62 (5.3)	0 (0.0)	0 (0.0)	
2013	12 (11.2)	0 (0.0)	75 (6.4)	0 (0.0)	0 (0.0)	
2014	12 (11.2)	0 (0.0)	99 (8.4)	0 (0.0)	0 (0.0)	
2015	12 (11.2)	0 (0.0)	105 (8.9)	41 (7.8)	5 (5.1)	
2016	6 (5.6)	0 (0.0)	125 (10.6)	56 (10.6)	13 (13.3)	
2017	19 (17.8)	1 (2.9)	116 (9.8)	120 (22.8)	32 (32.7)	
2018	14 (13.1)	9 (25.7)	137 (11.6)	149 (28.3)	29 (29.6)	
2019	15 (14.0)	18 (51.4)	244 (20.7)	160 (30.4)	19 (19.4)	
2020	7 (6.5)	7 (20.0)	216 (18.3)	0 (0.0)	0 (0.0)	
Conditioning regimen, *n* (%)						<.0001^b^
MAC	106 (99.1)	28 (80.0)	1019 (86.4)	450 (85.6)	70 (71.4)	
RIC	1 (0.9)	7 (20.0)	160 (13.6)	76 (14.4)	28 (28.6)	
ATG use, *n* (%)						<.0001^b^
Yes	2 (1.9)	0 (0.0)	754 (64.0)	10 (1.9)	23 (23.5)	
No	105 (98.1)	35 (100.0)	425 (36.0)	516 (98.1)	75 (76.5)	
aGVHD prophylaxis, *n* (%)						<.0001^b^
CsA + MMF	0 (0.0)	35 (100.0)	0 (0.0)	512 (97.3)	84 (85.7)	
CsA + MTX	0 (0.0)	0 (0.0)	390 (33.1)	0 (0.0)	0 (0.0)	
CsA + MMF + MTX	0 (0.0)	0 (0.0)	212 (18.0)	12 (2.3)	6 (6.1)	
TAC + MTX	0 (0.0)	0 (0.0)	292 (24.8)	0 (0.0)	0 (0.0)	
TAC + MMF + MTX	0 (0.0)	0 (0.0)	283 (24.0)	0 (0.0)	0 (0.0)	
Others	0 (0.0)	0 (0.0)	1 (0.1)	2 (0.4)	8 (8.1)	
Not used	107 (100.0)	0 (0.0)	1 (0.1)	0 (0.0)	0 (0.0)	
CD34^+^ cell dosage in 10^6^ cells/kg, median (range)	2.20 (0.50–10.24)	0.17 (0.03–0.71)	2.81 (1.04–19.76)	0.19 (0.01–1.06)	4.70 (1.26–16.19)	<.0001^a^
Post‐transplant immune profiling						.003 ^a^
Follow‐up duration, median (range)	1.24 (0.18–6.28)	0.81 (0.18–2.08)	1.32 (0.17–7.73)	1.22 (0.17–5.50)	1.46 (0.17–4.95)	
Had ≥1 immune profiles after 360 days post‐transplant, *n* (%)	68 (63.6)	13 (37.1)	770 (65.3)	323 (61.4)	69 (70.4)	.004 ^b^
Had ≥1 immune profiles after 720 days post‐transplant, *n* (%)	40 (37.4)	2 (5.7)	397 (33.7)	163 (31.0)	36 (36.7)	.006 ^b^
Had ≥1 immune profiles after 1080 days post‐ transplant, *n* (%)	20 (18.7)	0 (0.0)	187 (15.9)	82 (15.6)	12 (12.2)	.085 ^b^
Infection, *n* (%)						
Days 1–30 post‐transplant	53 (49.5)	22 (62.9)	257 (21.8)	123 (23.4)	9 (9.2)	<.0001^b^
Days 31–60 post‐transplant	2 (1.9)	5 (14.3)	199 (16.9)	49 (9.3)	2 (2.0)	<.0001^b^
Days 61–90 post‐transplant	3 (2.8)	4 (11.4)	79 (6.7)	34 (6.5)	0 (0.0)	.028 ^b^
Days 91–180 post‐transplant	2 (1.9)	4 (11.4)	108 (9.2)	64 (12.2)	8 (8.2)	.017 ^b^
Days 181–360 post‐transplant	3 (2.8)	3 (8.6)	124 (10.5)	68 (12.9)	4 (4.1)	.006 ^b^
Severe aGVHD within 100 days, *n* (%)						<.0001^b^
Yes	0 (0.0)	7 (20.0)	101 (8.6)	66 (12.5)	3 (3.1)	
No	107 (100.0)	28 (80.0)	1078 (91.4)	460 (87.5)	95 (96.9)	
Relapse‐related death, %						.608 ^c^
6‐month	0.9	0.0	0.5	1.1	1.0	
1‐year	3.8	2.9	3.6	4.0	3.1	
3‐year	14.5	9.4	9.5	9.2	8.3	
Non‐relapse mortality, %						.089 ^c^
6‐month	0.0	0.0	2.0	5.1	5.1	
1‐year	0.0	0.0	6.4	9.1	8.2	
3‐year	3.8	3.4	12.1	10.8	12.2	
Overall survival, %						.700 ^d^
6‐month	99.1	97.1	97.4	93.7	93.9	
1‐year	96.2	97.1	90.0	86.9	88.8	
3‐year	81.7	87.2	78.4	80.0	79.4	

Abbreviations: ALL, acute lymphoblastic leukemia; AML, acute myeloid leukemia; ATG, antithymocyte globulin; BM, bone marrow; BMF, bone marrow failure; CsA, cyclosporine A; Haplo, haploidentical; HLA, human leukocyte antigen; HSCT, hematopoietic stem cell transplantation; MAC, myeloablative conditioning; MDS, myelodysplastic syndromes; MMF, mycophenolate mofetil; MMUD, HLA‐mismatched unrelated donor; MSD, HLA‐matched sibling donor; MTX, methotrexate; MUD, HLA‐matched unrelated donor; PB, peripheral blood; RIC, reduced‐intensity conditioning; TAC, tacrolimus; UCB, umbilical cord blood.

^a^ Kruskal‐Wallis test.

^b^ Chi‐square test.

^c^ Fine‐Gray test.

^d^ Log‐rank test.

**FIGURE 1:**
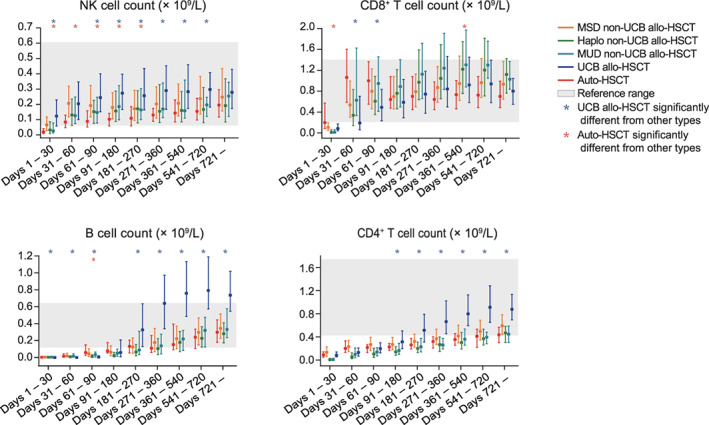
Population‐level univariate statistics of post‐transplant immune reconstitution. Shown are the post‐transplant profiles of natural killer (NK), CD8^+^ T, B, and CD4^+^ T cells in the auto‐hematopoietic stem cell transplantation (HSCT) (*n* = 107), matched sibling donor (MSD) non‐UCB (umbilical cord blood) allo‐HSCT (*n* = 729), haploidentical (Haplo) non‐UCB allo‐HSCT (*n* = 443), matched unrelated donor (MUD) non‐UCB allo‐HSCT (*n* = 89), and UCB allo‐HSCT (*n* = 561) cases. The 25th‐, 50th‐, and 75th‐percentile values for each subgroup of the patients during each time period are plotted. The red and blue asterisks indicate the time periods during which auto‐HSCT and UCB allo‐HSCT were significantly different from each of the other types of transplant (*p* < .05, pairwise two‐sided Wilcoxon rank‐sum test), respectively. There was no correction for multiple‐hypothesis testing. Mismatched unrelated donor (MMUD) non‐UCB allo‐HSCT is excluded here due to the small number of cases (*n* = 16). [Color figure can be viewed at wileyonlinelibrary.com]

At the population level, CD8^+^ T cell count rose slower in umbilical cord blood (UCB) allo‐HSCT than in the other types of HSCT. On the other hand, NK, CD4^+^ T, and B cell counts eventually reached higher levels in UCB allo‐HSCT than in the other types of transplantation (Figure 1). NK cell count rose slower in auto‐HSCT than in the other types of transplant (Figure 1).

**FIGURE 2:**
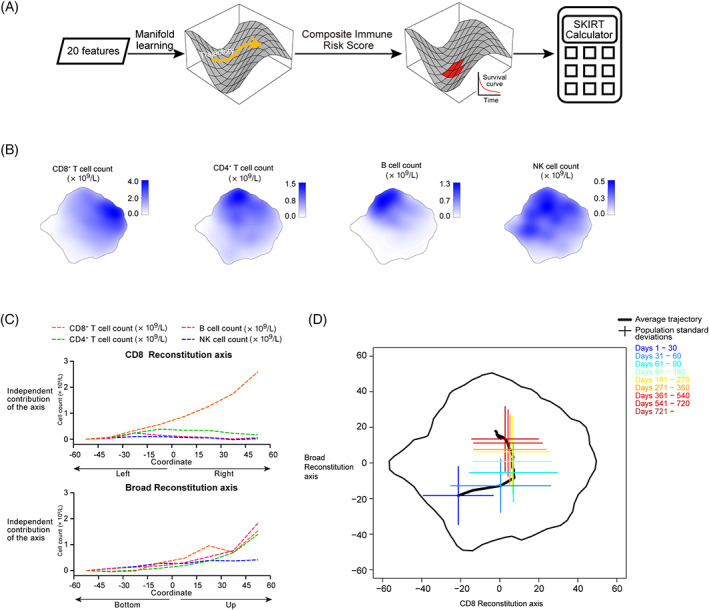
Visualizing the trajectory of post‐transplant immune reconstitution. (A) Analytic pipeline. (B) The gradients of CD8^+^ T, CD4^+^ T, B, and NK cell counts in the two‐dimensional phase space. The values of the clinical features are indicated by color intensity. (C) The gradients of NK, CD8^+^ T, CD4^+^ T, and B cell counts along the horizontal and vertical axes. Each plotted value represents how a given coordinate along one of the two axes independently contributed to one of the four clinical features. (D) The average trajectory of post‐transplant immune reconstitution across the entire Systems Kinetics of Immune Reconstitution Tianjin (SKIRT) dataset. The population standard deviations at selected landmark time periods are also plotted. Different time periods are indicated by different colors. [Color figure can be viewed at wileyonlinelibrary.com]

To advance beyond univariate descriptive statistics at the population level, we embedded the 11 150 immune profiles onto a two‐dimensional “phase space” (Figure 2A,B). We found that, along the horizontal axis of the two‐dimensional “phase space,” the phenotypic gradient was limited to primarily CD8^+^ T cell count; in contrast, along the vertical axis, there were broad‐ranged gradients in most of the immune cell counts (Figure 2C). As a consequence, we termed the horizontal axis the “CD8 Reconstitution” axis and the vertical axis the “Broad Reconstitution” axis.

On average, immune reconstitution evolved along the CD8 Reconstitution axis (that is, from left to right) during the initial two months after transplant, and afterward it evolved along the Broad Reconstitution axis (that is, from bottom to up) (Figure 2D). Embedding the immune data onto the two‐dimensional plane enabled us to compare the individual trajectories of immune reconstitution (Appendix, p. 5). The between‐patient variance of immune status peaked at ≈day 60 post‐transplant, and afterward, the patients became more uniform gradually (Figure 2D and Appendix, p. 6).

We then investigated if there was any clinical consequence when a patient deviated from the average trajectory of immune reconstitution. In other words, we inquired if there was any time‐dependent signature of immune reconstitution that was highly associated with overall survival after transplant (Figure 2A). We focused on allo‐HSCT in the ensuing analysis.

**FIGURE 3:**
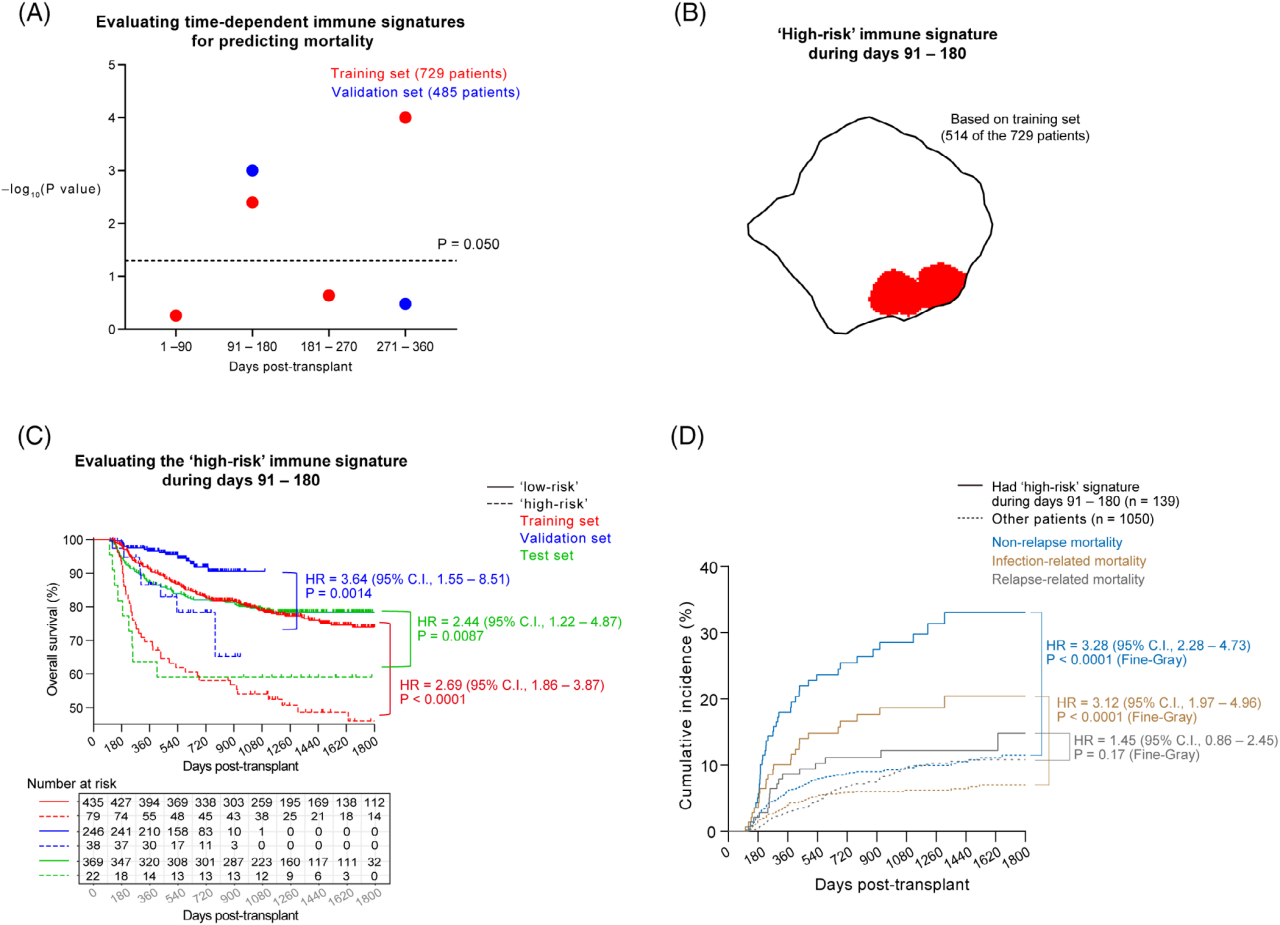
Discovery and verification of a Composite Immune Risk Score during days 91–180 for predicting early mortality. (A) A candidate composite immune signature associated with days 91–180 passed statistical significance for predicting early mortality in both the training and validation sets. The *p* values were calculated using Cox regression. The horizontal dotted line indicates *p* = .05. (B) The “high‐risk” composite immune signature during days 91–180 calculated based on the training‐set data. (C) Overall survival of the “high‐risk” and “low‐risk” patients in the training, validation, and test sets. Here the classification of the patients into “high‐risk” and “low‐risk” was based on the Composite Immune Risk Score during days 91–180, whose calculation formula was fitted solely based on the training‐set data. (D) The causes of death among the 1189 patients who had immune profiling data during days 91–180 post‐transplant. [Color figure can be viewed at wileyonlinelibrary.com]

For each of the four post‐transplant time periods (that is, days 1–90, days 91–180, days 181–270, and days 271–360), we tested if there existed a composite immune signature that predicted mortality using a ten‐fold cross‐validation approach within the training set. Both “days 91–180” and “days 271–360” passed statistical significance (*p* < 0.05, Cox regression) in the training set. Only the composite immune signature during days 91–180, however, passed statistical significance (*p* < 0.05, Cox regression) in both the training and validation sets (Figure 3A,B). (It should be noted that only 514 (70.5%) of the 729 training‐set patients had available immune profiling data during days 91–180, and only 284 (58.6%) of the 485 validation‐set patients had available data during days 91–180. There was no significant difference between the clinical course of the subsets of patients who had immune profiling data during days 91–180 and the clinical course of all the patients in the training set and the validation set (Appendix, pp. 1–2).)

The “high‐risk” composite immune signature during days 91–180 post‐transplant could be simplified as a scoring function by fitting a logistic regression model to the training set: Composite Immune Risk Score (during days 91–180 post‐transplant) = White blood cell count (10^9^/L) × 0.7304 + Neutrophil count (10^9^/L) × (−0.4745) + Lymphocyte percentage in nucleated cells (×100%) × (−16.7825) + Lymphocyte count (10^9^/L) × (−1.7612) + NK cell percentage in lymphocytes (×100%) × (−7.9450) + T cell percentage in lymphocytes (×100%) × 10.2955 + CD4^+^ T cell percentage in lymphocytes (×100%) × (−7.9703) + B cell percentage in lymphocytes (×100%) × (−15.7887). When the Composite Immune Risk Score was >2.50 during days 91–180, the patient was deemed “high‐risk.”

Seventy‐nine (15.4%) of the 514 training‐set patients and 38 (13.4%) of the 284 validation‐set patients were “high‐risk” according to the Composite Immune Risk Score during days 91–180. “High risk” during days 91–180 had hazard ratios (HR) of 2.69 (*n* = 514; 95% C.I., 1.86–3.87; *p* < .0001) and 3.64 (*n* = 284; 95% C.I., 1.55–8.51; *p* = .0014) for early mortality in the training and validation sets, respectively (Figure 3C).

We then tested the Composite Immune Risk Score in the hold‐out test set from the FAHUSTC. 391 (62.7%) of the 624 test‐set patients from the FAHUSTC had available post‐transplant immune profiling data during days 91–180. There was no significant difference between the clinical course of the subset of test‐set patients who had immune profiling data during days 91–180 and the clinical course of all the patients in the test set (Appendix, pp. 1–2).

Using the Composite Immune Risk Score, we stratified the 391 patients in the test set into “high‐risk” (score >2.50) and “low‐risk” (score ≤2.50) based on their immune profiles during days 91–180; 22 (5.6%) of the 391 patients turned out to be “high‐risk” during days 91–180. We found that the “high‐risk” patients were indeed much more likely to suffer from early mortality than the “low‐risk” patients (HR, 2.44 (95% C.I., 1.22–4.87); *p* = .0087; Figure 3C). Therefore, although the formula for the Composite Immune Risk Score was discovered in the IHCAMS cohort of patients that was primarily composed of non‐UCB transplants, a high Composite Immune Risk Score nonetheless proved to be predictive of early mortality in the UCB‐predominant FAHUSTC cohort also.

Altogether across the entire SKIRT dataset, when comparing the “high‐risk” patients against the “low‐risk” patients, the HR (computed by the Fine‐Gray model to correct for competing risks) for relapse‐related deaths, NRM, and infection‐related deaths were 1.45 (95% C.I., 0.86–2.45; *p* = .17), 3.28 (95% C.I., 2.28–4.73; *p* < .0001), and 3.12 (95% C.I., 1.97–4.96; *p* < .0001), respectively (Figure 3D); that is, the “high‐risk” patients were significantly more likely than the “low‐risk” patients to suffer from NRM.

**FIGURE 4:**
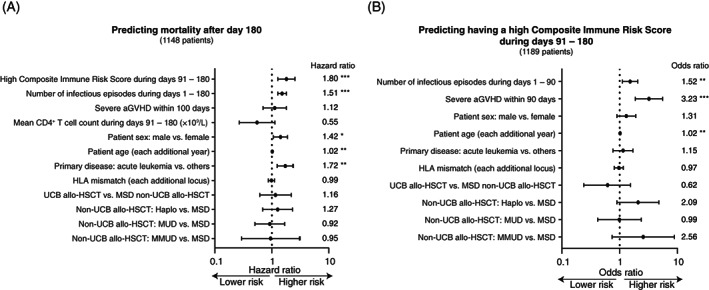
The Composite Immune Risk Score during days 91–180 independently predicted early mortality. (A) The factors that predicted mortality after (excluding) day 180. The multivariate model was fitted using the Cox regression method (*, *p* < .05; **, *p* < .01; ***, *p* < .001). “High‐risk” here was defined according to the Composite Immune Risk Score during days 91–180. (B) The factors that influenced the probability of having a high Composite Immune Risk Score during days 91–180. The multivariate model was fitted using the logistic regression method (*, *p* < .05; **, *p* < .01; ***, *p* < .001).

Even when other factors such as average CD4^+^ T cell count during days 91–180 (a predictor of clinical outcome that had stronger consensus among the experts based on previously available data^5^) were included as the covariates in the Cox regression model, a high Composite Immune Risk Score during days 91–180 remained an independent risk factor for early mortality after allo‐HSCT (HR, 1.80 (95% C.I., 1.28–2.55); *p* = .00085) (Figure 4A).

A high Composite Immune Risk Score during days 91–180 remained an independent predictor of mortality, even if we limited the multivariate analysis to the subset of all adult patients (HR, 1.86 (95% C.I., 1.30–2.66); *p* = .00062), adult patients with acute leukemia (HR, 1.63 (95% C.I., 1.07–2.50); *p* = .024), or adult patients with diseases other than acute leukemia (HR, 2.78 (95% C.I., 1.38–5.60); *p* = .0042) (Appendix, p. 7). In contrast, in the pediatric patients, average CD4^+^ T cell count during days 91–180 (but not the Composite Immune Risk Score) was an independent predictor for mortality (HR per 10^9^ cells/L increase, 0.07 (95% C.I., 0.01–0.70); *p* = .024) (Appendix, p. 7).

Severe aGVHD during the initial 90 days post‐transplant (odds ratio (OR), 3.23; 95% C.I., 1.87–5.58; *p* < .0001), the number of infectious episodes during the initial 90 days (OR per additional episode, 1.52; 95% C.I., 1.12–2.06; *p* = .0075), and patient age (OR per additional year, 1.02; 95% C.I., 1.01–1.04; *p* = .0034) were all risk factors for having high Composite Immune Risk Scores during days 91–180 after allo‐HSCT (Figure 4B).

## 4 DISCUSSION

To our knowledge, the present research is the largest cohort study of post‐HSCT immune reconstitution to date. Similar to the intent of numerous studies in the past,^1^, ^2^, ^3^, ^4^, ^5^, ^6^, ^7^, ^8^, ^9^, ^10^, ^19^, ^21^, ^23^, ^26^ we aimed to delineate the relationship between immune reconstitution and clinical outcome. Our study utilized unsupervised machine learning to identify the temporal patterns of multivariate immune variables after transplantation, which were then combined with survival modeling using grid‐search optimization to result in a Composite Immune Risk Score for predicting early mortality. We validated the Composite Immune Risk Score in two independent subsets of the patients from two medical centers. Despite the many differences between UCB‐ and peripheral blood‐derived stem cell transplants,^29^ the Composite Immune Risk Score is an independent prognostic factor for overall survival in allo‐HSCT in both the IHCAMS cohort (composed of predominantly peripheral blood‐derived transplants) and the FAHUSTC cohort (composed of predominantly UCB‐derived transplants). To facilitate the clinicians to visualize the trajectory of post‐HSCT immune reconstitution and to compute the Composite Immune Risk Score during days 91–180 post‐transplant, we have developed an online tool‐the SKIRT Calculator (https://skirt-calculator.shinyapps.io/skirt-calculator/) – that is publicly accessible to all.

In recent years, there has been growing interest in devising new computational methods for analyzing multidimensional phenotypes in diseases (for instance, acute respiratory distress syndrome,^30^ type 2 diabetes,^31^ aGVHD,^32^ and sepsis^33^). For post‐transplant immune reconstitution, Toor et al. have developed the methodology of using logistic dynamics to classify the temporal profiles of the total lymphocyte count post‐transplant into three distinct growth patterns,^21^ Koenig et al. have explored the application of principal component analysis to measure the distance between an HSCT patient's immune status and the immune status of non‐hematological patients and the use of this distance for predicting overall survival,^10^ and Mellgren et al. have proposed to use reflected discriminant analysis to decompose post‐transplant immune reconstitution into two independent axes (one for cell normalization and the other for functional maturation).^34^ It should be noted that none of these studies analyzed more than 50 cases of HSCT, conducted independent validation of the proposed methodology, or summarized their result into a scoring formula that can be easily applied at the clinic. In contrast, in search of a scoring function that is ideally as simple as the “body mass index” for assessing the degree of obesity, our study identified an easy‐to‐compute formula for the Composite Immune Risk Score that integrates eight immune factors for predicting early mortality after HSCT.

For long, it has been known that immune reconstitution after HSCT recapitulates aspects of immune ontogeny during development.^29^ There has been, however, no equivalent of a “growth chart” that can be used to quantitatively assess a patient's immune reconstitution over time after transplant. In this study, we used manifold learning to visualize the individual patients' trajectories of immune reconstitution and found that the population variance is the largest around day 60 post‐transplant. While most patients' immune reconstitution continues to evolve upward in the phase space after day 60, in some patients the trajectory becomes “derailed” and instead progresses rightward or even downward, entering a “high‐risk” territory that is characterized by high Composite Immune Risk Scores during days 91–180. Evidently the critical time point of day 60 plays a decisive role in the direction of immune reconstitution during the ensuing 1 to 4 months.

Our data also indicate that there are intricate relationships between infection and immune reconstitution. Infection is a risk factor for entering the “high‐risk” immune territory during days 91–180, and a high Composite Immune Risk Score in turn is an independent risk factor that predicts infection‐related death, that is, independent from earlier infectious episodes. For those patients whose Composite Immune Risk Scores are high during days 91–180, we submit that special attention needs to be paid to the prevention and control of infection, perhaps by intensified monitoring of the patients' vital signs and complete blood counts in the outpatient setting; alternatively, pharmaceutical or cytotherapeutic interventions could potentially be tested in these high‐risk patients to rectify their immune reconstitution (Appendix, p. 8). In contexts other than managing these high‐risk patients whose immune status has derailed during days 91–180, a few strategies of potential interventions have been studied for their ability to modulate post‐HSCT immune reconstitution,^5^, ^35^, ^36^, ^37^, ^38^ and some of these strategies might prove useful for improving the overall survival of the “high‐risk” patients.

Limited by the lack of data from normal children, we did not investigate the relationship between child development and immune reconstitution. It is, however, crucial to evaluate the patient's immune data in reference to the patient's age and the immune system's normal development.^39^, ^40^ We do provide evidence that the “high‐risk” immune signature might be different in children versus adults. In the subgroup analysis, we found that for adults the Composite Immune Risk Score during days 91–180 is a better prognostic factor for overall survival than CD4^+^ T cell count during days 91–180, while the opposite is true for children. Bertaina et al. have recently championed for concentrated effort to validate the prognostic value of CD4^+^ T cell count^5^; our study provides new data that support its use in pediatric patients of allo‐HSCT.

Future research directions could include prospective validation of the Composite Immune Risk Score in adult patients of allo‐HSCT, investigation of the relationship between child development and post‐transplant immune reconstitution, and the elucidation of the molecular and cellular mechanisms underlying the inter‐individual differences in immune reconstitution during days 60–180 post‐transplant.

## AUTHOR CONTRIBUTIONS

Yigeng Cao, Xiaowen Gong, Yahui Feng, Mingyang Wang, Yu Hu, Huilan Liu, Xueou Liu, Saibing Qi, Yanping Ji, and Fang Liu are the co‐first authors. Ye Guo, Zimin Sun, Erlie Jiang, and Junren Chen are the co‐senior authors. Junren Chen, Erlie Jiang, Zimin Sun, Ye Guo, and Xiaofan Zhu supervised the study. Junren Chen and Erlie Jiang designed the study, with contributions from Zimin Sun, Ye Guo, and Xiaofan Zhu. Xueou Liu, Junren Chen, and Zimin Sun led the effort for obtaining the ethics approvals, with contributions from Erlie Jiang and Xiaofan Zhu. Yigeng Cao and Xueou Liu coordinated the study, with contributions from Yu Hu and Mingyang Wang. Xiaowen Gong and Mingyang Wang accessed and verified the underlying data. Yigeng Cao, Xiaowen Gong, Yahui Feng, Mingyang Wang, Yu Hu, Huilan Liu, Xueou Liu Yanping Ji, Ye Guo, Huaiping Zhu, Junren Chen, and Zimin Sun curated the dataset, with contributions from Wenwen Guo. Junren Chen designed the algorithms, with contributions from Xiaowen Gong, Yahui Feng, and Saibing Qi. Xiaowen Gong, Yahui Feng, Saibing Qi, Xueou Liu, Yu Hu, and Junren Chen performed the analysis, with contributions from Qiujin Shen and Wei Zhang. Saibing Qi and Yu Hu built the online tool, with contributions from Xiaowen Gong, Yahui Feng, Qiujin Shen, Yigeng Cao, and Mingyang Wang. All the authors contributed to the interpretation of the results. Xueou Liu led the effort to ensure the public accessibility of the underlying data, with contributions from Xiaowen Gong and Yahui Feng. Xiaowen Gong, Yahui Feng, and Saibing Qi led the effort to ensure the public accessibility of the computational code. Junren Chen, Xiaowen Gong, Yahui Feng, Yigeng Cao, Xueou Liu, Mingyang Wang, Yu Hu, and Qiujin Shen wrote the manuscript, with contributions from Erlie Jiang, Zimin Sun, Ye Guo, Xiaofan Zhu, and Wei Zhang.

## CONFLICT OF INTEREST

The authors declare no conflict of interest.

## Supporting information


**Table S1.** No substantial difference between the clinical course of the subsets of patients who had immune profiling data during days 91–180 and the clinical course of all the patients in the training, validation, and test sets
**Figure S1.** Flowchart for patient selection
**Figure S2.** The temporal distribution of the post‐transplant immune profiling data in Systems Kinetics of Immune Reconstitution Tianjin
**Figure S3.** The trajectories of post‐transplant immune reconstitution in five example patients
**Figure S4.** Inter‐patient variance of immune status evolved over time
**Figure S5.** A high Composite Immune Risk Score during days 91–180 was an independent predictor for mortality in different subsets of the adult patients.
**Figure S6.** The use of the “Composite Immune Risk Score” in adult patients after allo‐hematopoietic stem cell transplantation

## Data Availability

This study did not involve the genetic information of any individual. The Chinese government approved (CJ0913 (2022)) the public sharing of the portion of the SKIRT dataset that originated from the IHCAMS (covering 8358 (75.0% of 11150) post‐transplant immune profiles from 1321 patients (67.9% of 1945) in SKIRT), and we have deposited the 1321 patients' raw, desensitized data at the National Genomics Data Center of the People's Republic of China (https://ngdc.cncb.ac.cn/omix/release/OMIX001396/). The processed, desensitized data of all the 1945 patients (that is, the peri‐transplant parameters and the coordinates of all the 11150 immune profiles in the two‐dimensional phase space) are available in a public Zenodo repository (https://doi.org/10.5281/zenodo.7317671). For 420 (21.6% of 1945) of the patients in SKIRT, the multivariate time‐series of ≥159 clinical features during the initial 100 days post‐transplant and their peri‐transplant characteristics such as patient age, patient sex, primary diagnosis, transplant type, and stem cell source have been published elsewhere.^32^ The computational code used in this study is available in a public GitHub repository (https://github.com/chenjunren-ihcams/SKIRT).

## References

[1] Storek J , Gooley T , Witherspoon RP , Sullivan KM , Storb R . Infectious morbidity in long‐term survivors of allogeneic marrow transplantation is associated with low CD4 T cell counts. Am J Hematol. 1997;54:131‐138. 9034287 10.1002/(sici)1096-8652(199702)54:2<131::aid-ajh6>3.0.co;2-y

[2] Berger M , Figari O , Bruno B , et al. Lymphocyte subsets recovery following allogeneic bone marrow transplantation (BMT): CD4+ cell count and transplant‐related mortality. Bone Marrow Transplant. 2008;41:55‐62. 17934532 10.1038/sj.bmt.1705870

[3] Fedele R , Martino M , Garreffa C , et al. The impact of early CD4+ lymphocyte recovery on the outcome of patients who undergo allogeneic bone marrow or peripheral blood stem cell transplantation. Blood Transfus. 2012;10:174‐180. 22337266 10.2450/2012.0034-11 PMC3320776

[4] Admiraal R , van Kesteren C , Jol‐van der Zijde CM , et al. Association between anti‐thymocyte globulin exposure and CD4+ immune reconstitution in paediatric haemopoietic cell transplantation: a multicentre, retrospective pharmacodynamic cohort analysis. Lancet Haematol. 2015;2:e194‐e203. 26688094 10.1016/S2352-3026(15)00045-9

[5] Bertaina A , Abraham A , Bonfim C , et al. An ISCT stem cell engineering committee position statement on immune reconstitution: the importance of predictable and modifiable milestones of immune reconstitution to transplant outcomes. Cytotherapy. 2022;24:385‐392. 35331394 10.1016/j.jcyt.2021.09.011

[6] Kim SY , Lee H , Han MS , et al. Post‐transplantation natural killer cell count: a predictor of acute graft‐versus‐host disease and survival outcomes after allogeneic hematopoietic stem cell transplantation. Clin Lymphoma Myeloma Leuk. 2016;16:527‐535 e522. 27375156 10.1016/j.clml.2016.06.013

[7] Minculescu L , Marquart HV , Friis LS , et al. Early natural killer cell reconstitution predicts overall survival in T cell‐replete allogeneic hematopoietic stem cell transplantation. Biol Blood Marrow Transplant. 2016;22:2187‐2193. 27664326 10.1016/j.bbmt.2016.09.006

[8] Hattori N , Saito B , Sasaki Y , et al. Status of natural killer cell recovery in day 21 bone marrow after allogeneic hematopoietic stem cell transplantation predicts clinical outcome. Biol Blood Marrow Transplant. 2018;24:1841‐1847. 29753837 10.1016/j.bbmt.2018.05.007

[9] Shima T , Miyamoto T , Kikushige Y , et al. Quantitation of hematogones at the time of engraftment is a useful prognostic indicator in allogeneic hematopoietic stem cell transplantation. Blood. 2013;121:840‐848. 23233661 10.1182/blood-2012-02-409607

[10] Koenig M , Huenecke S , Salzmann‐Manrique E , et al. Multivariate analyses of immune reconstitution in children after allo‐SCT: risk‐estimation based on age‐matched leukocyte sub‐populations. Bone Marrow Transplant. 2010;45:613‐621. 19701252 10.1038/bmt.2009.204

[11] Rocha V , Labopin M , Sanz G , et al. Transplants of umbilical‐cord blood or bone marrow from unrelated donors in adults with acute leukemia. N Engl J Med. 2004;351:2276‐2285. 15564544 10.1056/NEJMoa041469

[12] Storek J , Dawson MA , Storer B , et al. Immune reconstitution after allogeneic marrow transplantation compared with blood stem cell transplantation. Blood. 2001;97:3380‐3389. 11369627 10.1182/blood.v97.11.3380

[13] Kalwak K , Gorczyńska E , Toporski J , et al. Immune reconstitution after haematopoietic cell transplantation in children: immunophenotype analysis with regard to factors affecting the speed of recovery. Br J Haematol. 2002;118:74‐89. 12100130 10.1046/j.1365-2141.2002.03560.x

[14] Petersen SL , Ryder LP , Björk P , et al. A comparison of T‐, B‐ and NK‐cell reconstitution following conventional or nonmyeloablative conditioning and transplantation with bone marrow or peripheral blood stem cells from human leucocyte antigen identical sibling donors. Bone Marrow Transplant. 2003;32:65‐72. 12815480 10.1038/sj.bmt.1704084

[15] Schwinger W , Weber‐Mzell D , Zois B , et al. Immune reconstitution after purified autologous and allogeneic blood stem cell transplantation compared with unmanipulated bone marrow transplantation in children. Br J Haematol. 2006;135:76‐84. 16925797 10.1111/j.1365-2141.2006.06244.x

[16] Federmann B , Hägele M , Pfeiffer M , et al. Immune reconstitution after haploidentical hematopoietic cell transplantation: impact of reduced intensity conditioning and CD3/CD19 depleted grafts. Leukemia. 2011;25:121‐129. 20944677 10.1038/leu.2010.235

[17] Chang YJ , Zhao XY , Huo MR , et al. Immune reconstitution following unmanipulated HLA‐mismatched/haploidentical transplantation compared with HLA‐identical sibling transplantation. J Clin Immunol. 2012;32:268‐280. 22173879 10.1007/s10875-011-9630-7

[18] Jacobson CA , Turki AT , McDonough SM , et al. Immune reconstitution after double umbilical cord blood stem cell transplantation: comparison with unrelated peripheral blood stem cell transplantation. Biol Blood Marrow Transplant. 2012;18:565‐574. 21875503 10.1016/j.bbmt.2011.08.018 PMC3288552

[19] Bartelink IH , Belitser SV , Knibbe CAJ , et al. Immune reconstitution kinetics as an early predictor for mortality using various hematopoietic stem cell sources in children. Biol Blood Marrow Transplant. 2013;19:305‐313. 23092812 10.1016/j.bbmt.2012.10.010

[20] Puissant‐Lubrano B , Huynh A , Attal M , Blancher A . Evolution of peripheral blood T lymphocyte subsets after allogenic or autologous hematopoietic stem cell transplantation. Immunobiology. 2014;219:611‐618. 24721705 10.1016/j.imbio.2014.03.012

[21] Toor AA , Sabo RT , Roberts CH , et al. Dynamical system modeling of immune reconstitution after allogeneic stem cell transplantation identifies patients at risk for adverse outcomes. Biol Blood Marrow Transplant. 2015;21:1237‐1245. 25849208 10.1016/j.bbmt.2015.03.011 PMC4836381

[22] Park BG , Park CJ , Jang S , et al. Reconstitution of lymphocyte subpopulations after hematopoietic stem cell transplantation: comparison of hematologic malignancies and donor types in event‐free patients. Leuk Res. 2015;39:1334‐1341. 26422556 10.1016/j.leukres.2015.09.010

[23] Kurata K , Yakushijin K , Mizuno I , et al. Early lymphocyte recovery predicts clinical outcome after HSCT with mycophenolate mofetil prophylaxis in the Japanese population. Int J Hematol. 2018;108:58‐65. 29569120 10.1007/s12185-018-2437-z

[24] Salzmann‐Manrique E , Bremm M , Huenecke S , et al. Joint modeling of immune reconstitution post haploidentical stem cell transplantation in pediatric patients with acute leukemia comparing CD34(+)‐selected to CD3/CD19‐depleted grafts in a retrospective multicenter study. Front Immunol. 2018;9:1841. 30154788 10.3389/fimmu.2018.01841 PMC6102342

[25] Rocha V , Cornish J , Sievers EL , et al. Comparison of outcomes of unrelated bone marrow and umbilical cord blood transplants in children with acute leukemia. Blood. 2001;97:2962‐2971. 11342418 10.1182/blood.v97.10.2962

[26] Waller EK , Logan BR , Fei M , et al. Kinetics of immune cell reconstitution predict survival in allogeneic bone marrow and G‐CSF‐mobilized stem cell transplantation. Blood Adv. 2019;3:2250‐2263. 31345792 10.1182/bloodadvances.2018029892 PMC6693008

[27] van der Maas NG , von Asmuth EGJ , Berghuis D , et al. Modeling influencing factors in B‐cell reconstitution after hematopoietic stem cell transplantation in children. Front Immunol. 2021;12:684147. 34025685 10.3389/fimmu.2021.684147 PMC8138425

[28] Harris AC , Young R , Devine S , et al. International, multicenter standardization of acute graft‐versus‐host disease clinical data collection: a report from the Mount Sinai acute GVHD international consortium. Biol Blood Marrow Transplant. 2016;22:4‐10. 26386318 10.1016/j.bbmt.2015.09.001 PMC4706482

[29] Yanir A , Schulz A , Lawitschka A , Nierkens S , Eyrich M . Immune reconstitution after allogeneic Haematopoietic cell transplantation: from observational studies to targeted interventions. Front Pediatr. 2021;9:786017. 35087775 10.3389/fped.2021.786017 PMC8789272

[30] Calfee CS , Delucchi K , Parsons PE , Thompson BT , Ware LB , Matthay MA . Subphenotypes in acute respiratory distress syndrome: latent class analysis of data from two randomised controlled trials. Lancet Respir Med. 2014;2:611‐620. 24853585 10.1016/S2213-2600(14)70097-9 PMC4154544

[31] Nair ATN , Wesolowska‐Andersen A , Brorsson C , et al. Heterogeneity in phenotype, disease progression and drug response in type 2 diabetes. Nat Med. 2022;28:982‐988. 35534565 10.1038/s41591-022-01790-7

[32] Liu X , Cao Y , Guo Y , et al. Dynamic forecasting of severe acute graft‐versus‐host disease after transplantation. Nat Comput Sci. 2022;2:153‐159. 10.1038/s43588-022-00213-4 PMC10766514 38177449

[33] Adams R , Henry KE , Sridharan A , et al. Prospective, multi‐site study of patient outcomes after implementation of the TREWS machine learning‐based early warning system for sepsis. Nat Med. 2022;28:1455‐1460. 35864252 10.1038/s41591-022-01894-0

[34] Mellgren K , Nierop AFM , Abrahamsson J . Use of multivariate immune reconstitution patterns to describe immune reconstitution after allogeneic stem cell transplantation in children. Biol Blood Marrow Transplant. 2019;25:2045‐2053. 31247315 10.1016/j.bbmt.2019.06.018

[35] Mackall CL , Fry TJ , Bare C , Morgan P , Galbraith A , Gress RE . IL‐7 increases both thymic‐dependent and thymic‐independent T‐cell regeneration after bone marrow transplantation. Blood. 2001;97:1491‐1497. 11222398 10.1182/blood.v97.5.1491

[36] Min D , Taylor PA , Panoskaltsis‐Mortari A , et al. Protection from thymic epithelial cell injury by keratinocyte growth factor: a new approach to improve thymic and peripheral T‐cell reconstitution after bone marrow transplantation. Blood. 2002;99:4592‐4600. 12036893 10.1182/blood.v99.12.4592

[37] Fry TJ , Sinha M , Milliron M , et al. Flt3 ligand enhances thymic‐dependent and thymic‐independent immune reconstitution. Blood. 2004;104:2794‐2800. 15226184 10.1182/blood-2003-11-3789

[38] Sutherland JS , Spyroglou L , Muirhead JL , et al. Enhanced immune system regeneration in humans following allogeneic or autologous hemopoietic stem cell transplantation by temporary sex steroid blockade. Clin Cancer Res. 2008;14:1138‐1149. 18281548 10.1158/1078-0432.CCR-07-1784

[39] Comans‐Bitter WM , de Groot R , van den Beemd R , et al. Immunophenotyping of blood lymphocytes in childhood. Reference values for lymphocyte subpopulations. J Pediatr. 1997;130:388‐393. 9063413 10.1016/s0022-3476(97)70200-2

[40] Small TN , Papadopoulos EB , Boulad F , et al. Comparison of immune reconstitution after unrelated and related T‐cell‐depleted bone marrow transplantation: effect of patient age and donor leukocyte infusions. Blood. 1999;93:467‐480. 9885208

