# Supplementary material for: The Composite Immune Risk Score predicts overall survival after allogeneic hematopoietic stem cell transplantation: A retrospective analysis of 1838 cases
Source: Am J Hematol. 2023 Jan 2;98(2):309–21. doi: 10.1002/ajh.26792 (PMC10108217; doi:10.1002/ajh.26792)

**Supplementary Materials**

**The Composite Immune Risk Score predicts overall survival after allogeneic hematopoietic stem cell transplantation: a retrospective analysis of 1838 cases**

Yigeng Cao, MD^1,2,7^, Xiaowen Gong, MS^1,2,7^, Yahui Feng, MS^1,2,7^, Mingyang Wang, MD^1,2,7^, Yu Hu, MD^1,2,7^, Huilan Liu, MD^3,4,7^, Xueou Liu, PhD^1,2,7^, Saibing Qi, MS^1,2,7^, Yanping Ji, MD^5,6,7^, Fang Liu, MD^1,2,7^, Huaiping Zhu, PhD^4^, Wenwen Guo, MS^1,2^, Qiujin Shen, PhD^1,2^, Rongli Zhang, MD^1,2^, Ningning Zhao, BS^1,2^, Weihua Zhai, MD^1,2^, Xiaoqiang Song, BS^1,2^, Xin Chen, MS^1,2^, Liangquan Geng, MD^3^, Xia Chen, MD^1,2^, Xuetong Zheng, MS^1,2^, Qiaoling Ma, MS^1,2^, Baolin Tang, MD^3^, Jialin Wei, MS^1,2^, Yong Huang, MD^1,2^, Yuanyuan Ren, MD^1,2^, Kaidi Song, MS^3^, Donglin Yang, MD^1,2^, Aiming Pang, MD^1,2^, Wen Yao, MD^3^, Yi He, MD^1,2^, Yue Shang, MS^1,2^, Xiang Wan, MD^3^, Wei Zhang, MS^1,2^, Song Zhang, BS^1,2^, Guangyu Sun, MS^3^, Sizhou Feng, MD^1,2^, Xiaofan Zhu, MD^1,2^, Mingzhe Han, MD^1,2^, Zhen Song, MS^1,2^, Ye Guo, MD^1,2,8^, Zimin Sun, MD^3,4,8^, Erlie Jiang, MD^1,2,8^, Junren Chen, MD^1,2,8^

^1^ State Key Laboratory of Experimental Hematology, National Clinical Research Center for Blood Diseases, Haihe Laboratory of Cell Ecosystem, Institute of Hematology & Blood Diseases Hospital, Chinese Academy of Medical Sciences & Peking Union Medical College, 288 Nanjing Road, Heping District, Tianjin 300020, China.

^2^Tianjin Institutes of Health Science, Building 23, Huijincheng, Tuanbo West, Jinghai District, Tianjin 301600, China.

^3^ Department of Hematology, The First Affiliated Hospital of University of Science and Technology of China, No. 17 Lujiang Road, Baohe District, Hefei 230001, China.

^4^ Blood and Cell Therapy Institute, Division of Life Sciences and Medicine, Anhui Provincial Key Laboratory of Blood Research and Applications, University of Science and Technology of China, No. 1 Swan Lake Road, Government Culture New District, Hefei 230001, China.

^5^ Anhui Medical University, No. 81 Meishan Road, Shushan District, Hefei 230032, China.

^6^ Department of Hematology, Affiliated Hospital of Jiangsu University, No. 438 Jiefang Road, Jingkou District, Zhenjiang 212000, China.

^7^ Co-first authors.

^8^ Co-senior authors.

Correspondence should be addressed to: Junren Chen, Institute of Hematology, Chinese Academy of Medical Sciences, No. 288 Nanjing Road, Tianjin 300020, China. Phone: (86-22) 2390-9109; E-mail: [chenjunren@ihcams.ac.cn](mailto:chenjunren@ihcams.ac.cn)

**Contents:**

**Supplementary Table1**

Table S1: No substantial difference between the clinical course of the subsets of patients who had immune profiling data during days 91 – 180 and the clinical course of all the patients in the training, validation, and test sets.1

**Supplementary Figures3**

Figure S1: Flowchart for patient selection.3

Figure S2: The temporal distribution of the post-transplant immune profiling data in SKIRT.4

Figure S3: The trajectories of post-transplant immune reconstitution in five example patients.5

Figure S4: Inter-patient variance of immune status evolved over time.6

Figure S5: A high Composite Immune Risk Score during days 91 – 180 was an independent predictor for mortality in different subsets of the adult patients.7

Figure S6: The use of the ‘Composite Immune Risk Score’ in adult patients after allo-HSCT.8

**Supplementary Table**

**Table S1. No significant difference between the clinical course of the subsets of patients who had immune profiling data during days 91 – 180 and the clinical course of all the patients in the training, validation, and test sets.**

|  | **Training Set** | | |  | **Validation Set** | | |  | **Test Set** | |  |
| --- | --- | --- | --- | --- | --- | --- | --- | --- | --- | --- | --- |
|  | **All (n = 729)** | **Had immune profiles during days 91 – 180**  **(n = 514)** | **P**  **value** |  | **All (n = 485)** | **Had immune profiles during days 91 – 180 (n = 284)** | **P value** |  | **All (n = 624)** | **Had immune profiles during days 91 – 180 (n = 391)** | **P value** |
| Infection, n (%) |  |  |  |  |  |  |  |  |  |  |  |
| Days 1 – 30 post-HSCT | 166 (22.8) | 121 (23.5) | 0.80 |  | 113 (23.3) | 58 (20.4) | 0.40 |  | 132 (21.1) | 80 (20.5) | 0.85 |
| Days 31 – 60 post-HSCT | 114 (15.6) | 73 (14.2) | 0.54 |  | 90 (18.6) | 54 (19.0) | 0.95 |  | 51 (8.2) | 33 (8.4) | 0.97 |
| Days 61 – 90 post-HSCT | 56 (7.7) | 40 (7.8) | > 0.99 |  | 27 (5.6) | 12 (4.2) | 0.52 |  | 34 (5.4) | 17 (4.3) | 0.53 |
| Days 91 – 180 post-HSCT | 73 (10.0) | 45 (8.8) | 0.27 |  | 39 (8.0) | 18 (6.3) | 0.66 |  | 72 (11.5) | 53 (13.6) | 0.71 |
| Days 181 – 360 post-HSCT | 83 (11.4) | 56 (10.9) | > 0.99 |  | 44 (9.1) | 24 (8.5) | 0.85 |  | 72 (11.5) | 51 (13.0) | 0.50 |
| Severe aGVHD within 100 days, n (%) |  |  | 0.72 |  |  |  | 0.39 |  |  |  | 0.17 |
| Yes | 62 (8.5) | 40 (7.8) |  |  | 46 (9.5) | 21 (7.4) |  |  | 69 (11.1) | 32 (8.2) |  |
| No | 667 (91.5) | 474 (92.2) |  |  | 439 (90.5) | 263 (92.6) |  |  | 555 (88.9) | 359 (91.8) |  |
| Relapse-related death^†^, % |  |  | 0.57 |  |  |  | 0.96 |  |  |  | 0.79 |
| 6-month | 0.7 | 1.0 |  |  | 0.2 | 0.4 |  |  | 1.1 | 0.8 |  |
| 1-year | 4.3 | 4.1 |  |  | 2.6 | 2.6 |  |  | 3.9 | 4.1 |  |
| 3-year | 10.3 | 11.3 |  |  | 6.1 | 5.0 |  |  | 9.1 | 9.9 |  |
| Non-relapse mortality^†^, % |  |  | 0.86 |  |  |  | 0.80 |  |  |  | 0.55 |
| 6-month | 2.7 | 1.9 |  |  | 0.8 | 0.7 |  |  | 5.1 | 5.6 |  |
| 1-year | 8.5 | 8.6 |  |  | 2.5 | 2.2 |  |  | 9.0 | 10.2 |  |
| 3-year | 14.2 | 14.1 |  |  | 7.8 | 8.2 |  |  | 11.0 | 12.4 |  |
| Over survival^‡^, % |  |  | 0.61 |  |  |  | 0.82 |  |  |  | 0.51 |
| 6-month | 96.6 | 97.1 |  |  | 99.0 | 98.9 |  |  | 93.7 | 93.6 |  |
| 1-year | 87.2 | 87.3 |  |  | 94.8 | 95.2 |  |  | 87.2 | 85.7 |  |
| 3-year | 75.3 | 74.6 |  |  | 86.0 | 86.8 |  |  | 79.9 | 77.7 |  |

^†^ Calculated using the Fine-Gray model to correct for competing events

^‡^ Calculated using the Kaplan-Meier method

Abbreviations: HSCT, hematopoietic stem cell transplantation; aGVHD, acute graft-versus-host disease.

**
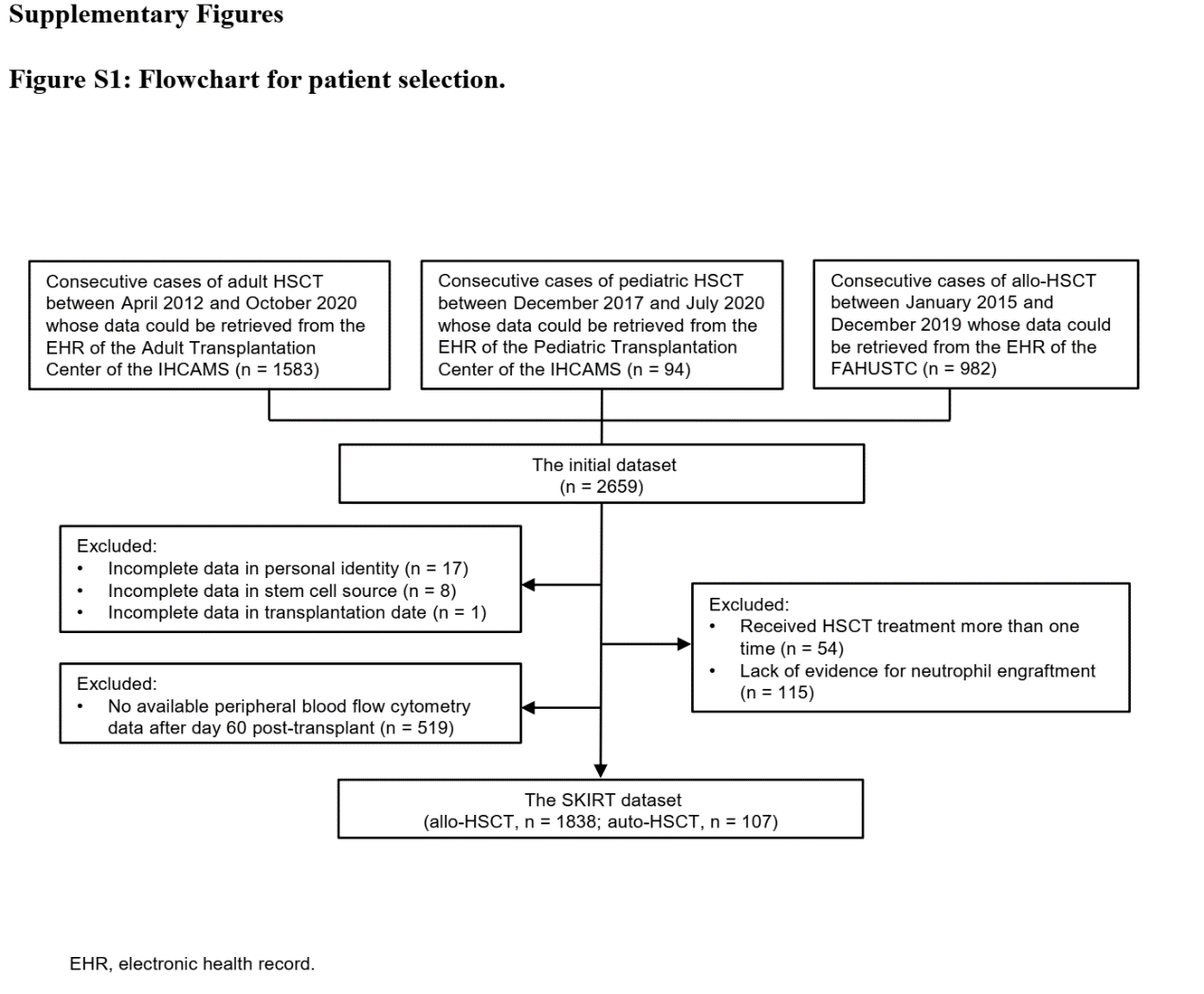
**


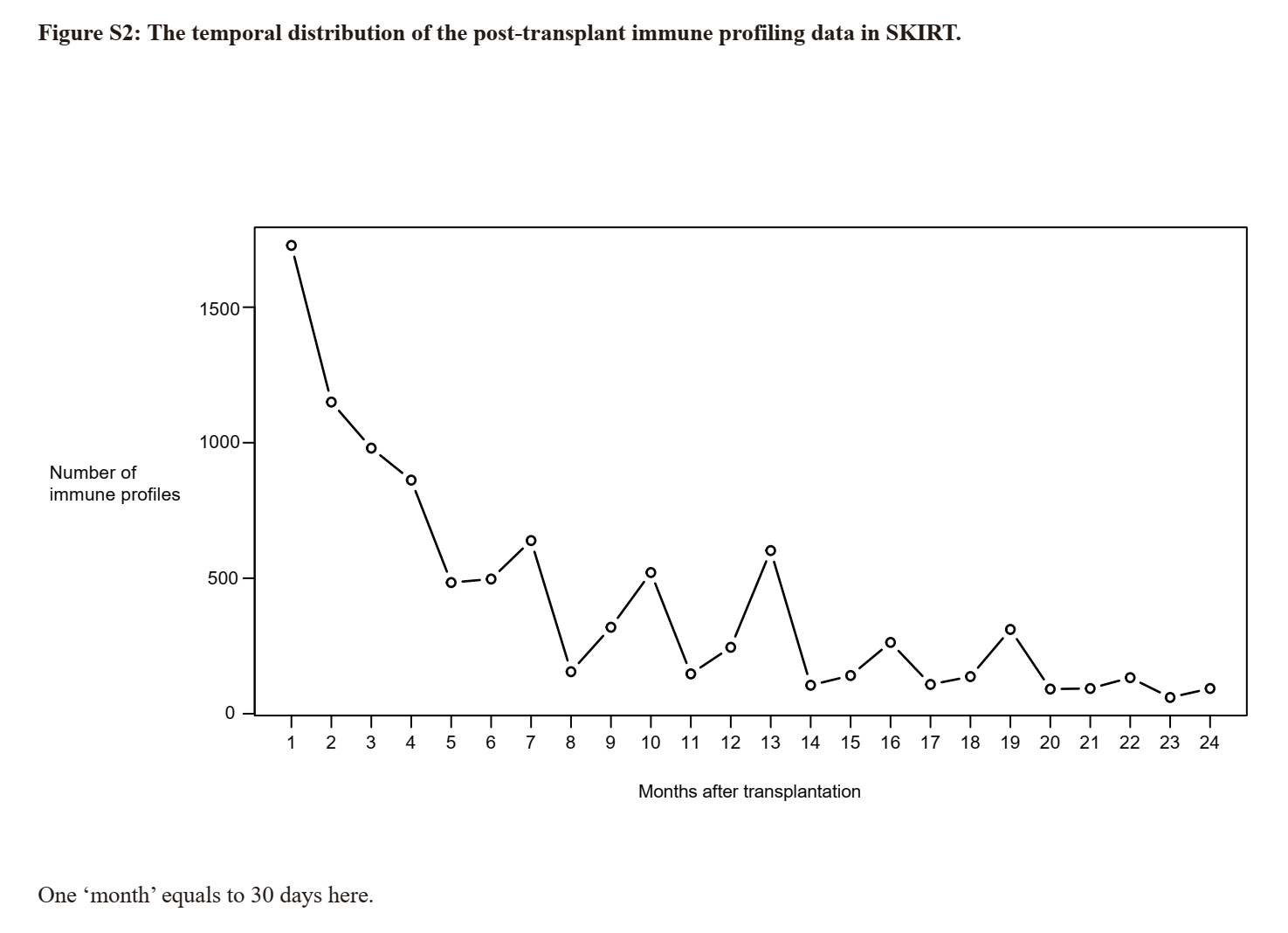


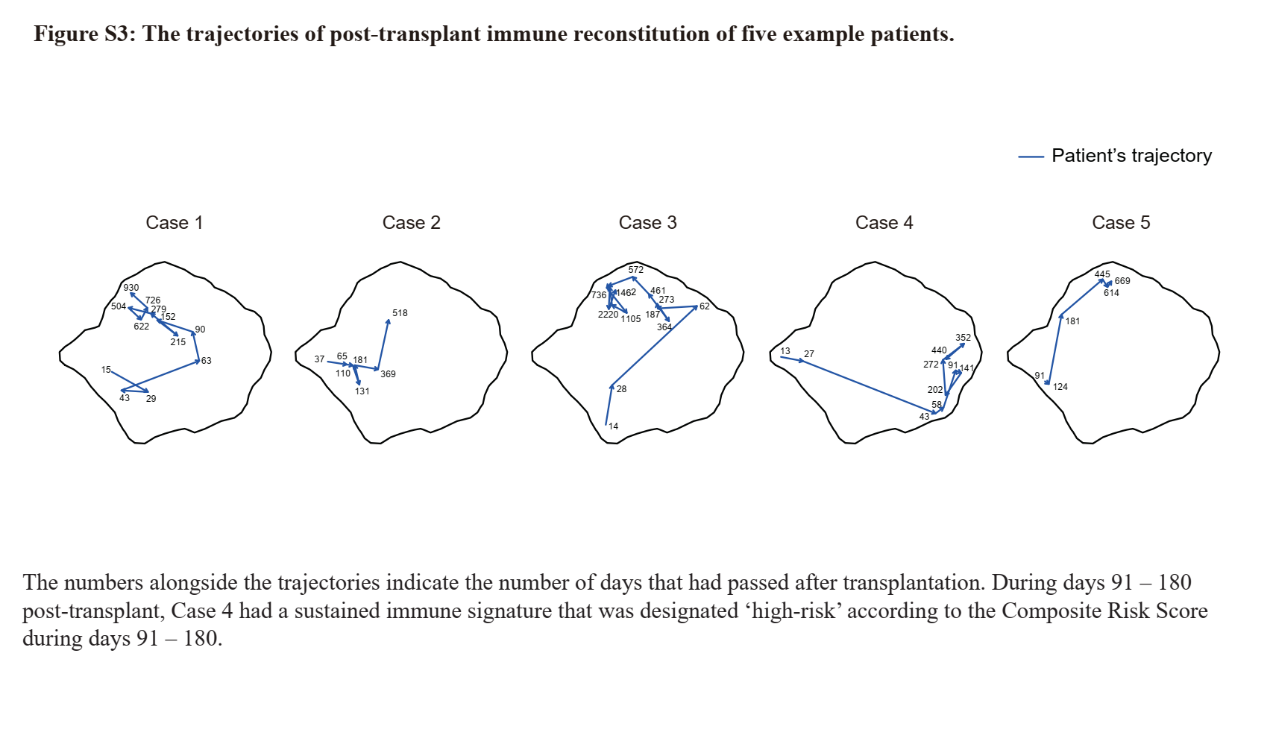


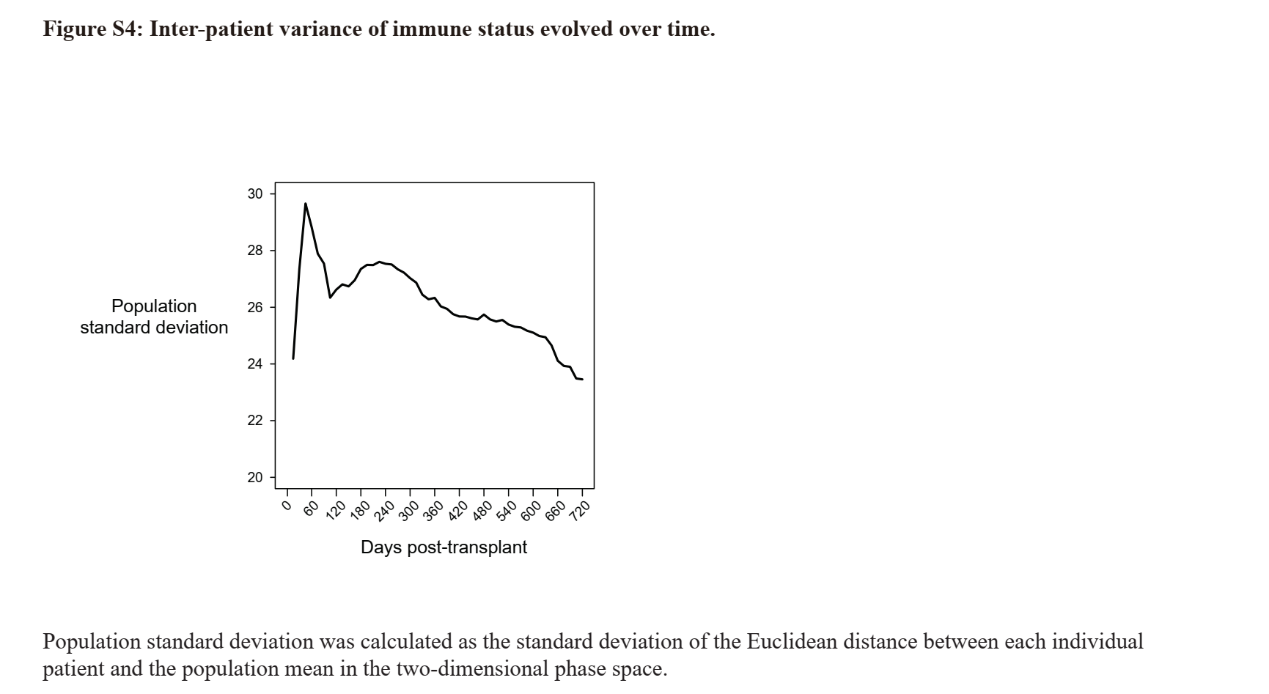


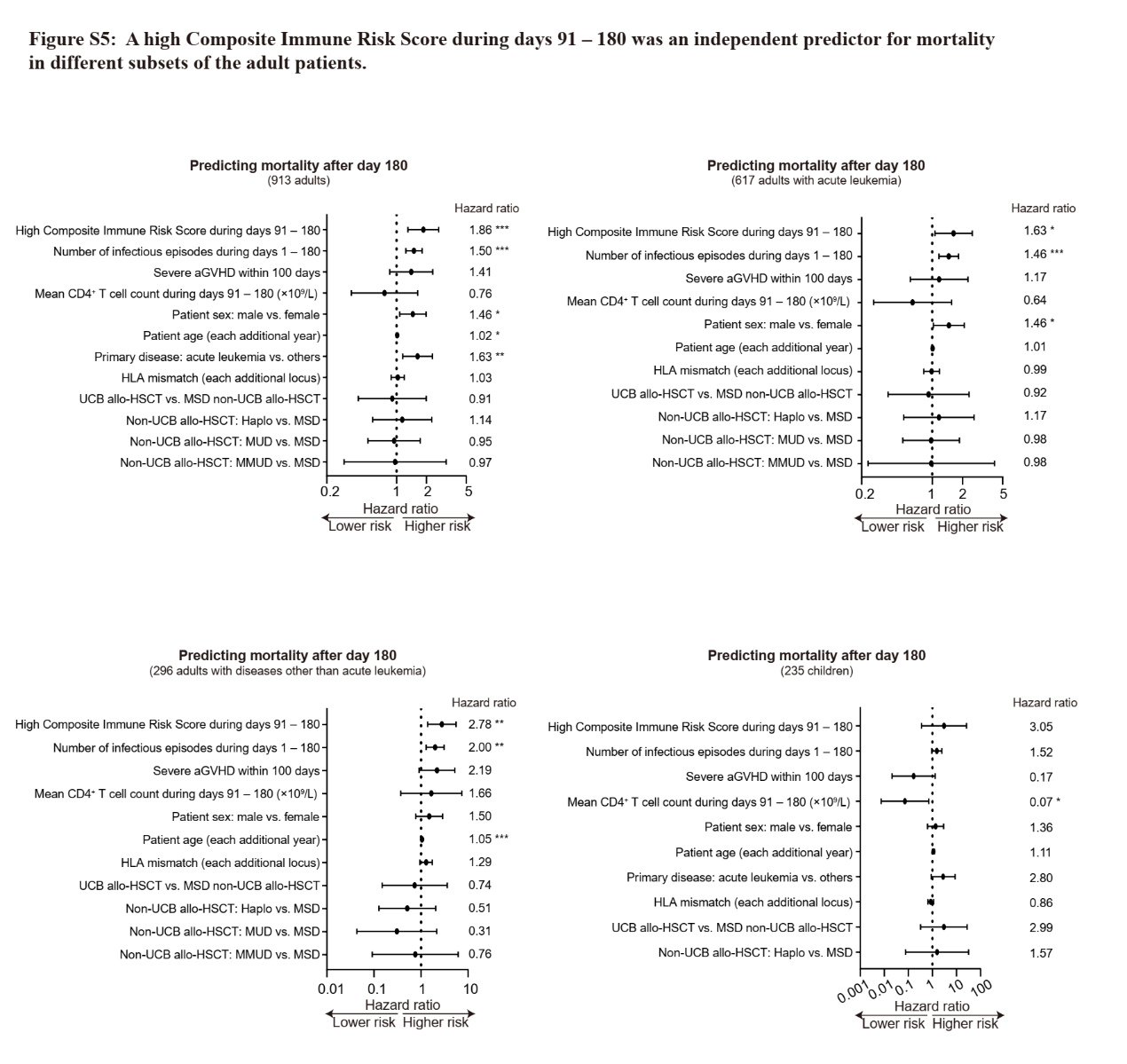


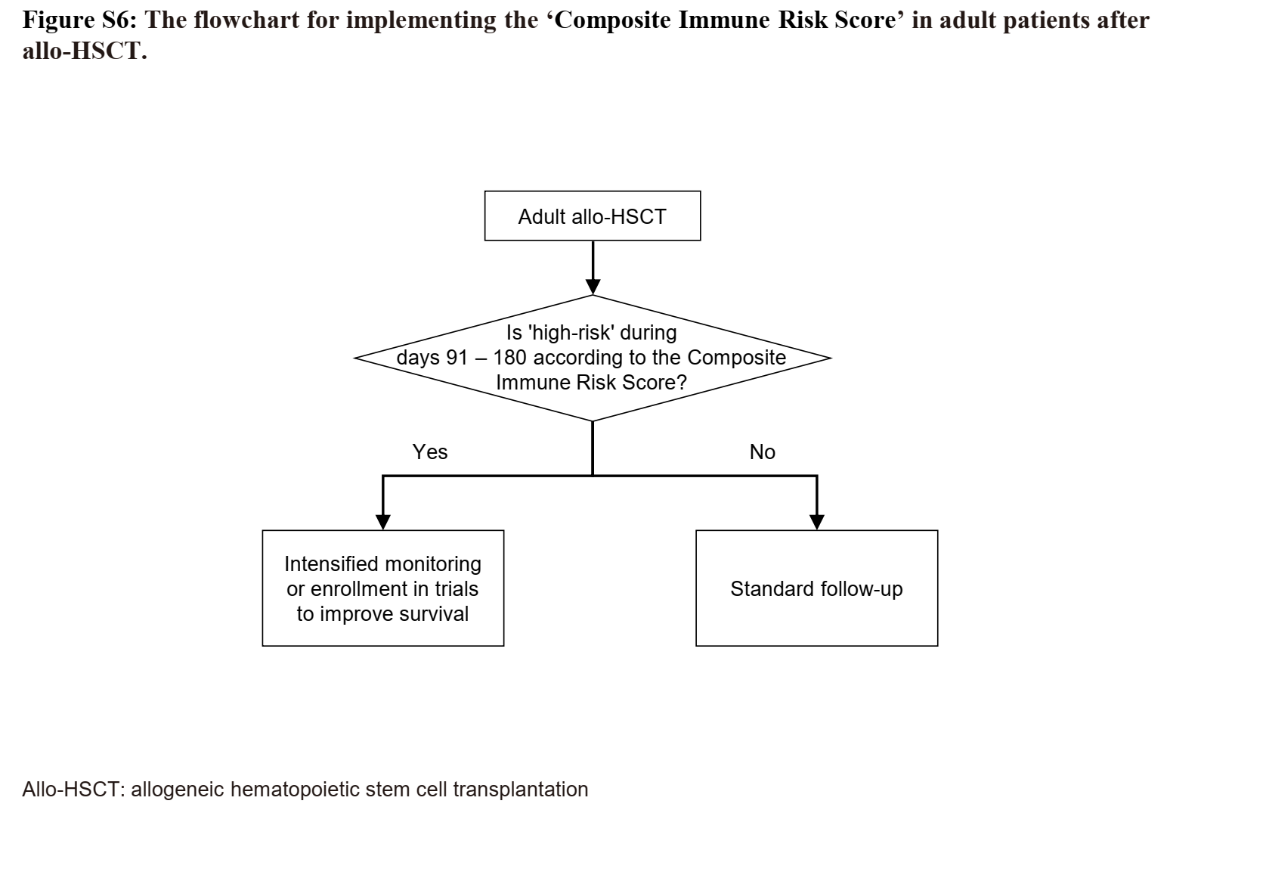

Supplement: Supplementary file 1 — Table S1. No substantial difference between the clinical course of the subsets of patients who had immune profiling data during days 91–180 and the clinical course of all the patients in the training, validation, and test sets Figure S1. Flowchart for patient selection Figure S2. The temporal distribution of the post‐transplant immune profiling data in Systems Kinetics of Immune Reconstitution Tianjin Figure S3. The trajectories of post‐transplant immune reconstitution in five example patients Figure S4. Inter‐patient variance of immune status evolved over time Figure S5. A high Composite Immune Risk Score during days 91–180 was an independent predictor for mortality in different subsets of the adult patients. Figure S6. The use of the “Composite Immune Risk Score” in adult patients after allo‐hematopoietic stem cell transplantation [file AJH-98-309-s001.docx]
